# Non-destructive analysis of a mixed H_2_O–CO_2_ fluid in experimental noble-metal capsule by means of freezing and high-energy synchrotron X-ray diffraction

**DOI:** 10.1038/s41598-022-24224-3

**Published:** 2022-11-24

**Authors:** Simone Tumiati, Marco Merlini, Andrea Amalfa, Marco Di Michiel, Luca Toffolo

**Affiliations:** 1 Dipartimento di Scienze Della Terra, Università Degli Studi di Milano, Milan, Italy; 2 European Synchrotron Radiation Facility, Grenoble, France

**Keywords:** Geochemistry, Petrology, Mineralogy

## Abstract

High-pressure high-temperature syntheses that involve volatile-bearing aqueous fluids are typically accomplished by enclosing the samples in gas-tight welded shut noble-metal capsules, from which the bulk volatile content must be extracted to be analyzed with mass spectroscopy, hence making the analysis non-replicable. Here we describe a novel non-destructive method that ensures the identification and the quantitative estimate of the volatiles directly in the sealed capsule, focusing on fluid H_2_O–CO_2_ mixtures equilibrated with graphite at conditions of geological interest (1 GPa, 800 °C). We used a high-energy (77 keV) synchrotron X-ray radiation combined with a cryostat to produce X-ray diffraction patterns and X-ray diffraction microtomographic cross-sections of the volatile-bearing samples down to –180 °C, thus encompassing the conditions at which crystalline phases-solid CO_2_ and clathrate (CO_2_ hydrate)-form. The uncertainty of the method is < 15 mol%, which reflects the difference between the volatile proportion estimated by both Rietveld refinement of the diffraction data and by image analysis of the microtomograms, and the reference value measured by quadrupole mass spectrometry. Therefore, our method can be reliably applied to the analysis of frozen H_2_O–CO_2_ mixtures and, moreover, has the potential to be extended to experimental fluids of geological interest containing other volatiles, such as CH_4_, SO_2_ and H_2_S.

## Introduction

Carbon dioxide, the main Earth’s greenhouse gas, is cycled between the solid Earth and the atmosphere over long timescales (> 1 Ma) by a variety of natural processes. Diffuse degassing and volcanic emissions are the final link in a long chain of devolatilization reactions occurring in the Earth’s interior^1^. The oxidation of the organic carbonaceous matter (soon transformed into graphitic carbon) and the decarbonation of sedimentary carbonate minerals^2^ in the subducted lithosphere are probably the most important processes supplying CO_2_ to the deep carbon cycle. This has been shown for instance in high-pressure high-temperature experiments reproducing the environmental conditions where CO_2_-releasing reactions are thought to occur at depth^3–5^. Recent advances in the synthesis and analysis of CO_2_-bearing aqueous fluids produced by oxidation of graphite at controlled redox conditions allowed to explore a wide range of compositional systems. In the C–O–H, the most simple system describing carbon-bearing aqueous fluids, it has been demonstrated that the composition of fluids in equilibrium with pure graphite is consistent with thermodynamic predictions^6–8^. However, an unpredictable oxidation behavior of graphite has been described in more complex systems containing contain silicates^3^, carbonates ^5^, disordered forms of graphite^6^ or disordered redox-buffering materials (e.g., NiO^8^). In these experimental studies, the redox state during the fluid synthesis has invariably been constrained using the so-called double-capsule technique^9^, which has been proven to be robust and reliable. Double capsules consist of an inner welded shut capsule, usually made of a noble-metal alloy permeable to hydrogen (e.g., Pt- or Pd-bearing alloys) and containing the experimental charge, and an outer welded shut capsule containing a redox-buffering mineral assemblage (usually metal + metal oxide or metal oxide + metal silicate, where the metal has different oxidation states) soaked in water. During the run at high pressure and temperature, redox reactions in the buffering mineral assemblage coupled with the dissociation of water constrain the *f*O_2_ and the *f*H_2_ in the outer capsule. Because of the permeability to H_2_, the same *f*H_2_ is expected in the inner capsule as well. When dealing with graphite oxidation, *f*H_2_ controls the amount of CO_2_ produced in the inner capsule containing graphite and water (see "Methods").

The composition of the final fluid is typically measured by puncturing the inner experimental capsule, conveying the volatiles towards a quadrupole mass spectrometer^7^, so that the fluid phase is eventually lost and unavailable for replicated analyses. Moreover, to set up the instrument for quantitative analyses, the species forming the fluid (e.g., H_2_O, CO_2_, CO, CH_4_) must be established in advance. In this study, we will describe a non-destructive method to qualitatively and quantitatively analyze the fluids contained in a noble-metal experimental capsule that relies on freezing the sample and analyzing it by X-ray diffraction, using a high-energy synchrotron source. This is made possible due to the fact that the solid H_2_O–CO_2_ mixture is crystalline when the temperature is below the freezing points of all the species that compose the fluid^10^. We will show that the 77 keV X-ray source, available at the ID15A line of the European Synchrotron Radiation Facility (ESRF; Grenoble, France), can effectively be used to obtain diffraction data and micro-tomographic cross sections of the inner capsule containing graphite and a H_2_O–CO_2_ fluid. We will then compare the results with the conventional (destructive) quadrupole mass spectrometry analysis of the fluid to estimate the reliability of our method.

## Methods

### Synthesis of fluids and sample preparation

Mixed H_2_O–CO_2_ fluids were generated by oxidation of graphite powder (obtained from spectroscopic pure rods gently hand-ground in boron carbide mortar) in ultrapure MilliQ water. Experiments were buffered using the double-capsule technique^9^ to prevent the direct contact of the sample with the buffering assemblages. An inner H_2_-permeable Au_60_Pd_40_ capsule (outer diameter OD = 2.3 mm; inner diameter ID = 2.0 mm; length ≈ 8 mm) and an outer Au capsule (OD = 3 mm; ID = 2.8 mm) were used. The outer capsule contained the redox buffer fayalite–magnetite–quartz (FMQ) soaked in water, constraining *f*H_2_. Quartz (SiO_2_) was obtained from powdered natural crystals; fayalite (Fe_2_SiO_4_) and magnetite (Fe_3_O_4_) have been synthesized at 1100 °C in a gas-mixing furnace in reducing atmosphere (CO_2_:CO = 10:1), starting from stoichiometrically weighted reagent-grade Fe_2_O_3_ (Sigma-Aldrich) and amorphous SiO_2_ obtained from hydrolyzed tetraethyl orthosilicate (Sigma-Aldrich). At equilibrium conditions, as long as all the buffer phases are present – which has been confirmed by scanning electron microscopy in almost identical runs performed at the same P–T conditions^3,6^ – the chemical potential of hydrogen is expected to be homogeneous in the inner and in the outer capsules. In the outer capsule, the hydrogen fugacity (*f*H_2_) is constrained through the reactions:

1 $${\text{3Fe}}_{{2}} {\text{SiO}}_{{4}} \left( {{\text{fayalite}}} \right) + {\text{2H}}_{{2}} {\text{O}} = {\text{2Fe}}_{{3}} {\text{O}}_{{4}} \left( {{\text{magnetite}}} \right) + {\text{3SiO}}_{{2}} \left( {{\text{quartz}}} \right) + {\text{2H}}_{{2}} \left( {{1}\;{\text{GPa}},{8}00\;^\circ {\text{C}}} \right)$$

In the inner capsule, the equilibration of the COH fluid is accomplished by the *f*H_2_-dependent reaction^3^:

2 $${\text{C}}\left( {{\text{graphite}}} \right) + {\text{2H}}_{{2}} {\text{O}} = {\text{CO}}_{{2}} + {\text{2H}}_{{2}}$$

As a consequence, the initial CO_2_-free aqueous fluid adjusts its CO_2_/H_2_O fraction until the equilibrium with the *f*H_2_ imposed by the buffer is reached. In a similar fashion, the oxygen fugacity (*f*O_2_) is constrained directly in the outer capsule by the FMQ buffer and indirectly in the inner capsule because of the water dissociation reaction:

3 $${\text{2H2O}} = {\text{2H2}} + {\text{O2}}$$

In the inner capsule, however, the *f*O_2_ will be slightly lower compared to that imposed by the FMQ buffer in the outer capsule since the fluid is not pure H_2_O but a H_2_O–CO_2_ mixture, with a consequent declined fugacity for H_2_O (and O_2_)^11^.

Experiments were performed at 1 GPa at 800 °C using an end-loaded piston-cylinder apparatus. Capsules were embedded in MgO rods (Norton Ceramics) and inserted in graphite furnaces surrounded by NaCl and borosilicate glass (Pyrex®). At the top of the assembly, a pyrophyllite–steel plug was placed to ensure the electrical contact. Temperatures were measured with K-type thermocouples that have an estimated uncertainty of ± 5 °C. An alumina disk was placed at the top of the capsule to avoid the direct contact with the thermocouple. Pressure calibration of the apparatus is based on the quartz to coesite transition^12^ that guarantees an uncertainty of ± 0.01 GPa. Samples were first brought to the run pressure, then heated to 800 °C, with a ramp of 100 °C/min. Run duration was 92 h, in order to approach equilibrium between graphite and CO_2_-bearing aqueous fluid at 800°C^3^. Eventually, the experiments were quenched by turning off the power supply, resulting in a cooling rate of > 40 °C/sec.

### X-ray diffraction of the frozen fluid

**Figure 1:**
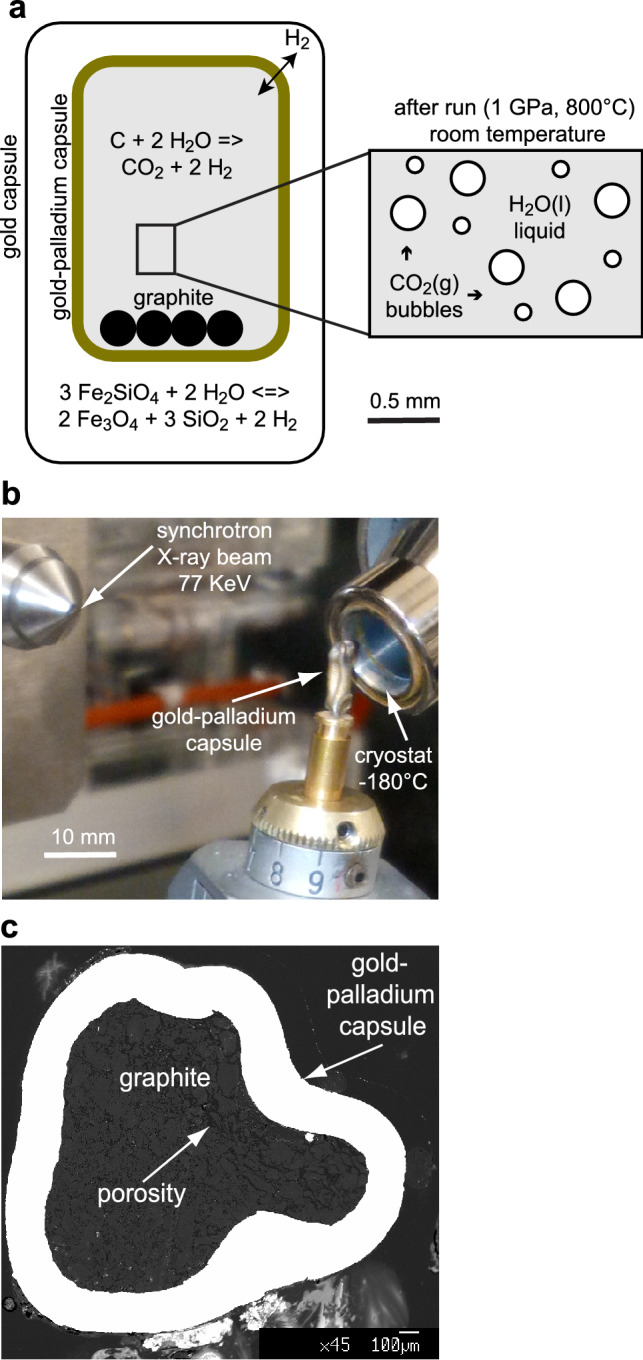
Experimental and analytical setup. (**a**) Sketch of the experimental capsule, consisting of an outer gold capsule containing an *f*H_2_–*f*O_2_ buffering assemblage (FMQ = fayalite + magnetite + quartz + water) and an inner gold–palladium capsule (brown thick line), permeable to hydrogen, containing graphite + water. At run conditions of 1 GPa, 800 °C and *f*H_2_^FMQ^, the oxidation of graphite produces CO_2_. (**b**) The inner gold–palladium capsule containing graphite and a mixed H_2_O–CO_2_ fluid is mounted on a goniometer head and cooled down to − 180 °C to allow the fluid to freeze into solid phases; X-ray diffraction effects inside the noble-metal capsule are collected using a high-energy 77 keV synchrotron source at ID15A of the ESRF. (**c**) Back-scattered electron image of a cross section of the inner capsule (white), mounted in epoxy resin and polished after synchrotron and QMS measurements; the former presence of a fluid phase can be inferred from the porosity between the graphite grains.

**Figure 2:**
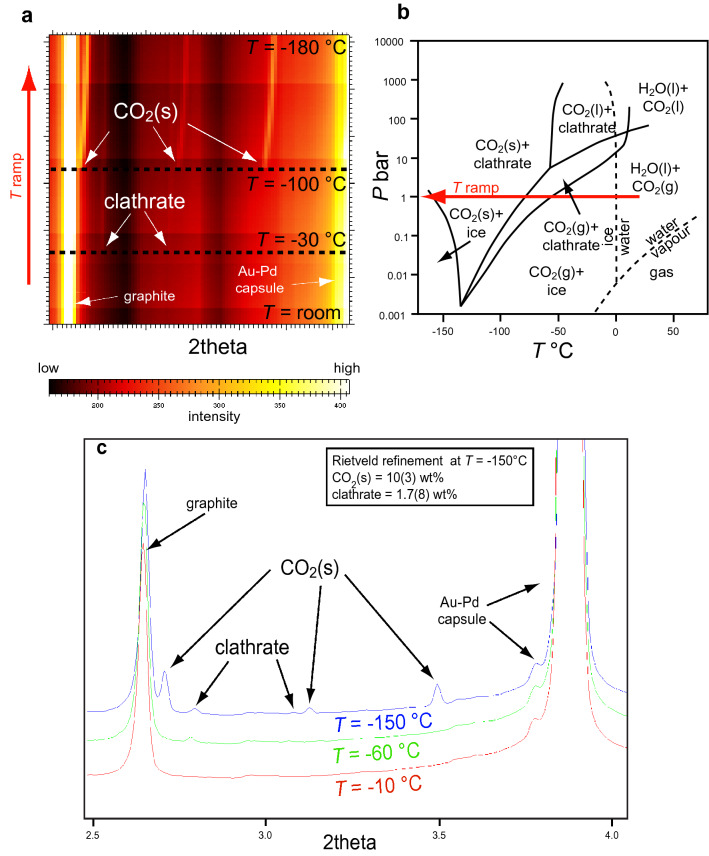
X-ray diffraction data collected during the cooling ramp from room temperature down to − 180 °C. (**a**) Clathrate and solid CO_2_ diffraction effects are detected below − 30 °C and − 100 °C, respectively. (**b**) Low-temperature phase diagram for the H_2_O–CO_2_ system redrawn after^10^; the experimental cooling ramp is shown by a red arrow. (**c**) Representative X-ray diffraction patterns collected at − 10 °C, − 60 °C and − 150 °C; the Rietveld refinement of the − 150 °C pattern yields 10 ± 3 wt% CO_2(solid)_ and 1.7 ± 0.8 wt% clathrate (a 100 wt% closure is imposed on the sum of CO_2(solid)_ + clathrate + graphite).

The inner Au–Pd capsule (Fig. 1a,b) containing graphite and H_2_O–CO_2_ fluid (Fig. 1c) was exposed by peeling off the outer gold capsule and removing the redox buffer (FMQ), then mounted on a goniometer head on the sample stage at the beamline ID15A of the ESRF (Fig. 1b). A Debye transmission geometry was used, and the signal was collected with a Dectris Pilatus3 X 2 M detector with a Cd–Te sensor. The beam had an energy of 77 keV (λ = 0.1610 Å) and a spot size of 0.05 × 0.05 mm on the sample. Such an elevated beam energy coupled with a high energy threshold of the detector minimized the absorption effects from the capsule (Au X-ray absorption edge is located at ~ 80.7 keV) and allowed a significant reduction of the background (mainly arising from Au_60_Pd_40_ fluorescence), respectively. The X-ray diffraction effects were collected every 2 min while cooling the sample with a cryostat from room temperature down to − 90 °C and from − 90 to − 180 °C with rates of − 3 °C/min and − 6 °C/min, respectively (Fig. 2a). The beam was focused orthogonally to the axis of the rotating capsule and multiple diffraction pattern collections were made at different z-axis positions. For each temperature, multiple scans with z offsets of 0.05 mm in 1 mm z interval were collected. Phase abundances were quantitatively estimated by performing Rietveld refinement and the proportions obtained from multiple acquisitions were used to calculate average values and the related uncertainties. Rietveld analysis was performed with GSAS + EXPGUI software^13,14^. Crystal structure of graphite, solid CO_2_, ice and clathrate were taken from ICSD database. Background, phase scale factors, lattice parameters and two peak profile function parameters (the constant Gaussian term and a term for Lorentian of a pseudo-Voigt function), were refined.

**Figure 3:**
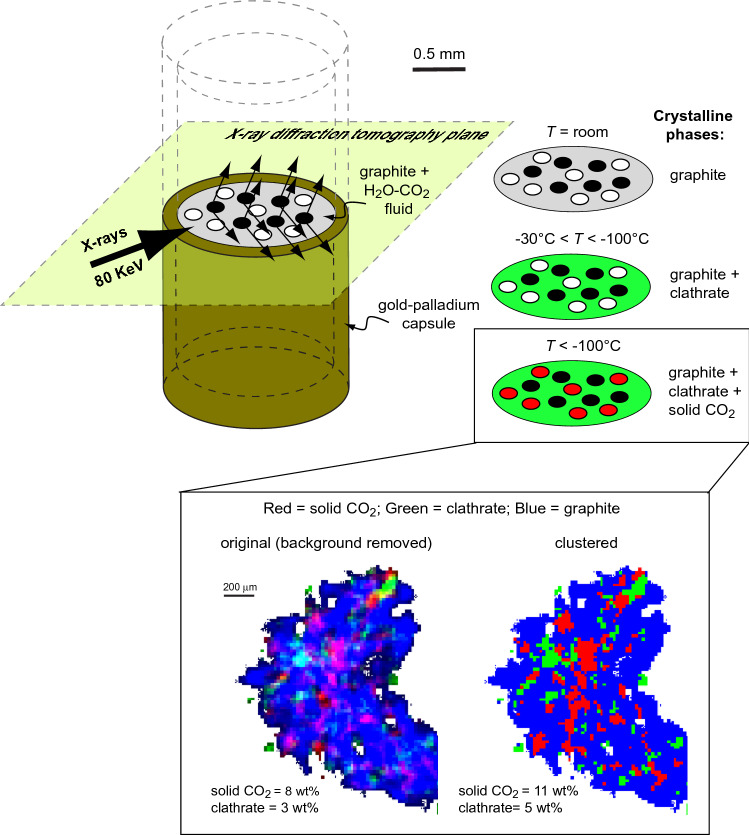
Schematic representation of X-ray diffraction microtomography of the experimental capsule (left) and the phase transitions observed in the H_2_O–CO_2_ fluid upon cooling (right). The inset shows the microtomogram collected at − 180 °C, i.e., when the fluid is converted into solid crystalline clathrate and CO_2(s)_: red, green and blue channels have been attributed to the characteristic peaks of solid CO_2_, clathrate and graphite, respectively, and combined into an RGB image after background removal (left) or clustering (right); the estimated mass percentages of solid CO_2_ and clathrate are reported.

X-ray diffraction microtomography^15^ was used to acquire a cross section of the middle part of the capsule at T = − 180 °C (Fig. 3), well below the freezing points of both H_2_O and CO_2_. An RGB image showing the spatial distribution of the phases in the capsule was obtained by assigning to the color channels the characteristic peaks of solid CO_2_, clathrate and graphite (red, green and blue, respectively, in Fig. 3; cf. also Supplementary Fig. 1). Background removal was applied to eliminate noise. Pixels of the RGB image were also classified using a K-means clustering method (Wolfram Mathematica).

### Quadrupole mass spectrometry

The capsule-piercing technique^7^ was employed for the analysis of quenched volatiles: the inner capsule was first punctured in vacuum conditions in a Teflon® reactor maintained at T ≈ 90 °C (at this temperature the liquid water contained in the sample evaporates completely); then, the gases were conveyed by an ultrapure Ar flux to an EXTORR 0–200 amu, Mod. XT200, quadrupole mass spectrometer (QMS), equipped with a secondary electron multiplier. The pressure and temperature conditions in the reactor were monitored by means of high-resolution pressure gauges (with a precision of ± 1 mbar) and a K-type thermocouple, respectively. Gas mixtures of known composition and ultrapure water were utilized as standards for the calibration of the QMS. The stability and the reproducibility of the analyses over time were monitored by periodically analyzing the volatile products released by thermal decomposition at 250 °C of 1 mg oxalic acid dihydrate contained in test capsules^7^. In this study the technique enabled to quantify micromolar quantities of the volatiles H_2_O and CO_2_ with uncertainties of 1.2% and 0.45%, respectively (Supplementary Table 1).

## Results

By cooling the sample, the volatiles contained in the capsule, composed of a mixture of water and gaseous CO_2_, crystallized into a mixture of clathrate (CO_2_·*n*H_2_O) and solid CO_2_ (Fig. 2b). From room temperature to − 30 °C, only the peaks of graphite and of the Au–Pd alloy are detectable in the diffraction patterns; at T = − 30 °C the peaks of a phase ascribable to clathrate appear and at T ≤ –100 °C they coexist with the peaks of solid CO_2_. The diffraction patterns remain almost unchanged down to − 180 °C, with the only exception of a subtle shift of the solid CO_2_ peaks towards higher diffraction angles. Apart from a slightly anticipated crystallization of water to clathrate (predicted at T ≈ − 60 °C) and a slightly retarded crystallization of CO_2(gas)_ to solid CO_2_ (predicted at T ≈ − 80 °C), our findings agree with proposed phase diagrams in the H_2_O–CO_2_ system^10^ (Fig. 2b), assuming that the pressure in the capsule is close to room conditions (red arrow in Fig. 2b). Representative X-ray diffraction patterns, collected at T = − 10 °C, − 60 °C and − 150 °C, are displayed in Fig. 2 c. Rietveld analyses showed slight inhomogeneities in the relative abundance of solid CO_2_ and clathrate (Supplementary Table 1), which can be averaged to 10 ± 3 wt% and 1.7 ± 0.8 wt%, respectively.

Microtomography of a single cross-section shows the micron-scale distribution in the capsule of the formerly volatile phases (Fig. 3); clathrate and solid CO_2_ fill the open porosity between graphite grains. By counting the average RGB pixel values, where R = solid CO_2_, G = clathrate and B = graphite, the volume percentages of solid CO_2_ and clathrate are estimated to 10 vol% and 6 vol%, respectively. Mass percentages are then calculated to be 8 wt% CO_2(solid)_ and 3–4 wt% clathrate, by considering that the density of solid CO_2_ is 1.68 g/cm^3^, while that of clathrate is in the range 1.18–1.36 g/cm^3^, depending on whether *n* is assumed to be 5.75 or 7^16^. A second way to estimate volume percentages is by means of image analysis (clustering), which allows to attribute every pixel of the microtomogram to a single phase. With this method, the volume percentages of solid CO_2_ and clathrate are 14 vol% and 9 vol%, respectively, which correspond to mass percentages of 11 wt% CO_2(solid)_ and 5–6 wt% clathrate.

### Validation of the method

Destructive QMS analysis of the volatiles extracted from the experimental capsule provides the reference values for the abundances of CO_2_ and H_2_O to be compared with the estimates obtained from X-ray diffraction of the frozen fluid. Mass spectrometry measurements give 12.66 ± 0.01 μmol CO_2(gas)_ and 7.80 ± 0.02 μmol H_2_O_(vapour)_. By considering the presence of 7.87 mg of graphite in the capsule, these values can be converted to mass percentages: 6.50 ± 0.03 wt% CO_2_ and 1.64 ± 0.02 wt% H_2_O, respectively, with a CO_2_/(H_2_O + CO_2_)_mass_ = 0.80 ± 0.05. A complication arises when the mass percentages of solid CO_2_ and clathrate derived by both Rietveld refinement of X-ray diffraction patterns and image analysis of the microtomogram are compared with the mass percentages of CO_2_ and H_2_O measured by QMS. In fact, while solid CO_2_ can be assumed to be almost pure because of the reported negligible content of H_2_O, clathrate (although dominated by H_2_O) is characterized by variable H_2_O/CO_2_ ratios, with H_2_O ranging from 5.75 to 7 mol per mole of CO_2_^10,16^. CO_2_ and H_2_O recalculated from the abundances of clathrate and solid CO_2_ are therefore: (i) 11 ± 0.03 wt% and 1.0 (*n* = 5.75)–1.1 (*n* = 7) ± 0.5 [CO_2_/(CO_2_ + H_2_O) ≈ 0.9], when Rietveld-refinement data are considered; (ii) 9–13 wt% and 2–4 wt% [CO_2_/(CO_2_ + H_2_O) ≈ 0.8], using micro-tomography data.

**Figure 4:**
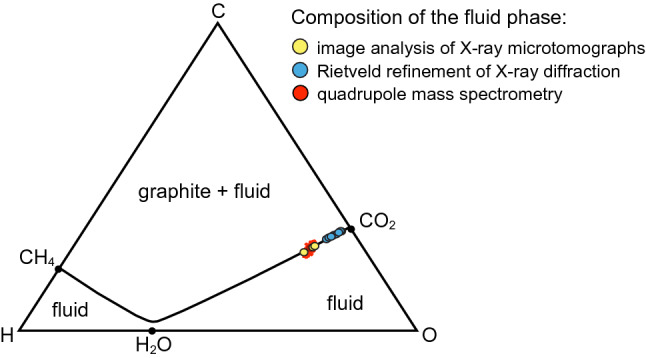
Fluid composition estimates (in wt%) plotted on a ternary C–O–H phase diagram (calculated at 1 GPa and 800 °C). The results of Rietveld refinement of single X-ray z-scans (blue dots) and image analysis (yellow dots) are compared with the reference fluid composition measured by QMS (red cloud). The black thick line represents all the possible compositions of graphite-saturated C–O–H fluids.

In Fig. 4 it is shown the comparison between the CO_2_/(H_2_O + CO_2_) mass ratios obtained from X-ray diffraction (blue dots), QMS (red cloud) and image analyses (yellow dots). Following this approach, the volatile components proportion from microtomogram analysis [CO_2_/(H_2_O + CO_2_) ~ 0.8] is consistent to that from QMS (0.80 ± 0.05) within uncertainties. Rietveld refinement of X-ray diffraction data, considering spatial variability, low absolute content of clathrate ≈ 1 wt%, possible uncertainties related to clathrate structure, and especially possible non randomly oriented crystallite and/or large crystal grains, all critical parameters in quantitative analysis^17,18^, provide also consistent values from 0.85 to 0.95: the average value of ~ 0.92 is only 15% higher than the reference QMS value.

## Conclusions

We demonstrate that high-energy synchrotron X-rays can be used to analyze – qualitatively and quantitatively-a frozen fluid phase sealed into a noble-metal experimental capsule. Temperatures below − 100 °C are required to freeze the H_2_O + CO_2_ fluid mixture into crystalline solid CO_2_ and clathrate. The total amount of fluid obtained both by (i) Rietveld refinement of X-ray diffraction patterns (12 ± 3.5 wt%) and (ii) image analysis of the X-ray diffraction microtomographic Sect. (11–17 wt%) agree within uncertainties with mass spectrometry measurements (8.14 ± 0.05 wt%). This protocol, tested for H_2_O–CO_2_ mixtures, is likely suitable for all those fluid species that can be frozen using a cryostat. For instance, methane (freezing point − 182 °C), sulfur dioxide (− 72 °C) and hydrogen sulfide (− 85.5 °C) could be suitable species of geological interest^19^. Our results open new perspectives when non-destructive analysis of experimental fluids is required.

## Supplementary Information

Supplementary Information.

## Data Availability

The authors declare that all data are available within the manuscript.
