# Supplementary material for: Non-destructive analysis of a mixed H2O–CO2 fluid in experimental noble-metal capsule by means of freezing and high-energy synchrotron X-ray diffraction
Source: Sci Rep. 2022 Nov 24;12:20240. doi: 10.1038/s41598-022-24224-3 (PMC9691697; doi:10.1038/s41598-022-24224-3)
Supplement: Supplementary file 1 — Supplementary Information. [file 41598_2022_24224_MOESM1_ESM.pdf]

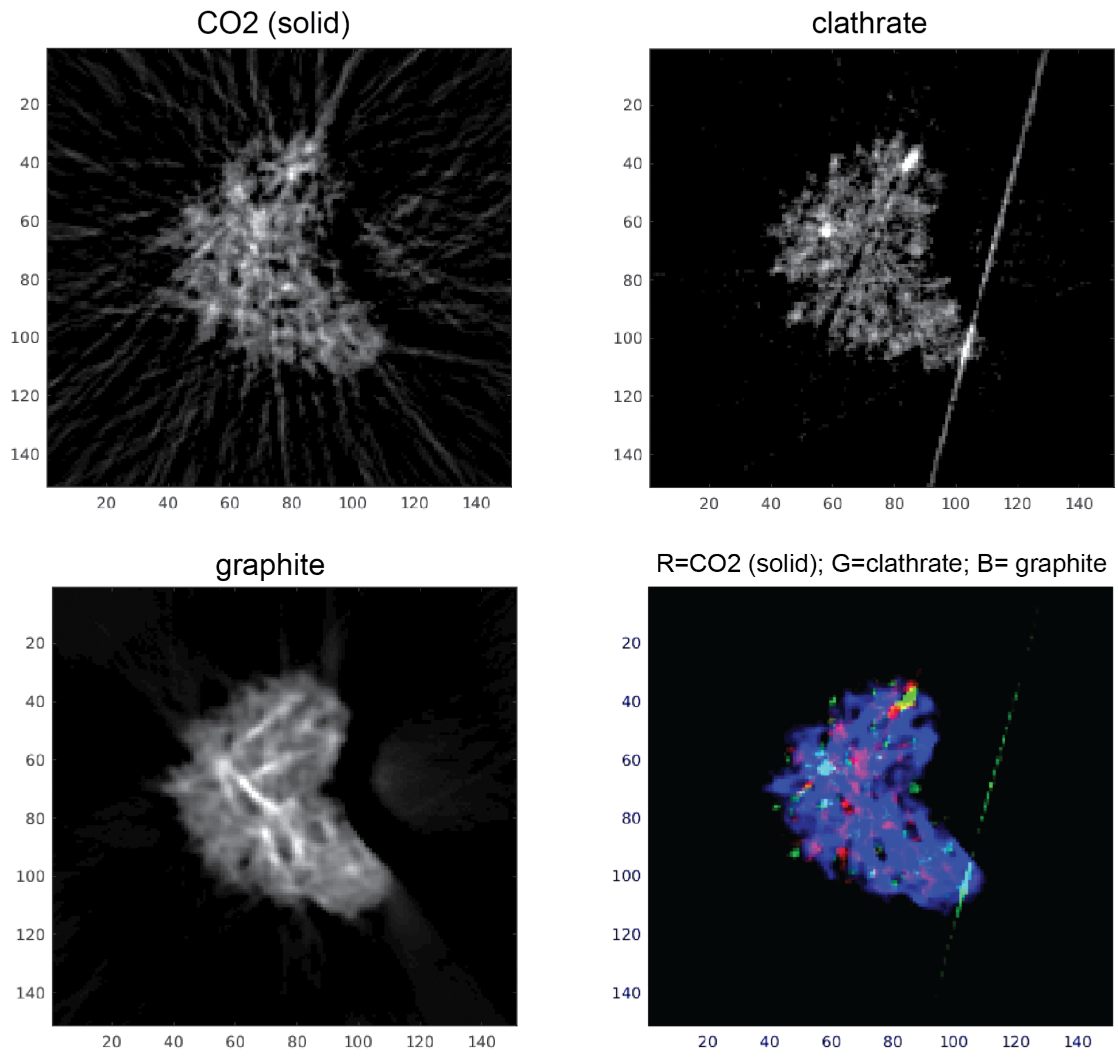

Supplementary Figure 1: X-ray diffraction microtomograms of solid CO_2, clathrate and graphite, and combined RGB image.

Supplementary Table 1: Analysis of volatiles.

	CO_2(solid) wt%	clathrate wt%	CO_2 wt%	H_2O wt%	$CO_2/(CO_2+H_2O)$ mass fraction
X-ray diffraction					
n = 5.75					
scan_50 μm	7.6	1.9	8.5	1.1	0.88
scan_100 μm	12.9	2.5	14.0	1.4	0.91
scan_150 μm	14.4	2.3	15.4	1.3	0.92
scan_200 μm	13.2	1.9	14.0	1.1	0.93
scan_250 μm	12.0	1.5	12.6	0.9	0.93
scan_300 μm	9.6	1.2	10.1	0.7	0.94
scan_350 μm	8.4	0.9	8.8	0.5	0.95
scan_400 μm	7.1	0.7	7.4	0.4	0.95
scan_450 μm	6.9	0.6	7.2	0.4	0.95
scan_500 μm	6.5	0.7	6.8	0.4	0.95
scan_550 μm	6.7	0.8	7.0	0.4	0.94
scan_600 μm	10.2	1.2	10.7	0.7	0.94
scan_650 mm	11.5	1.7	12.2	0.9	0.93
scan_700 μm	12.9	2.4	13.9	1.4	0.91
scan_750 μm	15.5	3.1	16.8	1.8	0.90
scan_800 μm	14.7	3.1	16.0	1.8	0.90
scan_850 μm	7.6	1.9	8.4	1.1	0.89
average	10(3)	1.7(8)	11(3)	1.0(5)	0.92(2)
n = 7					
scan_50 μm	7.6	1.9	8.3	1.3	0.87
scan_100 μm	12.9	2.5	13.8	1.6	0.89
scan_150 μm	14.4	2.3	15.3	1.5	0.91
scan_200 μm	13.2	1.9	13.9	1.3	0.92
scan_250 μm	12.0	1.5	12.5	1.0	0.93
scan_300 μm	9.6	1.2	10.0	0.8	0.93
scan_350 μm	8.4	0.9	8.7	0.6	0.94
scan_400 μm	7.1	0.7	7.3	0.4	0.94
scan_450 μm	6.9	0.6	7.1	0.4	0.95
scan_500 μm	6.5	0.7	6.8	0.4	0.94
scan_550 μm	6.7	0.8	6.9	0.5	0.93
scan_600 μm	10.2	1.2	10.6	0.8	0.93
scan_650 mm	11.5	1.7	12.1	1.1	0.92
scan_700 μm	12.9	2.4	13.7	1.5	0.90
scan_750 μm	15.5	3.1	16.5	2.0	0.89
scan_800 μm	14.7	3.1	15.7	2.0	0.89
scan_850 μm	7.6	1.9	8.3	1.2	0.87
average	10(3)	1.7(8)	11(3)	1.1(5)	0.91(3)
X-ray tomography					
RGB; n = 5.75	8	3	9	2	0.83
RGB; n = 7	8	4	9	2	0.79
clustering; n = 5.75	11	5	13	3	0.82
clustering; n = 7	11	6	13	4	0.78
average	10(2)	5(1)	11(2)	3(1)	0.81(2)
mass specrometry	-	-	6.50(3)	1.64(2)	0.80(5)
